# Exercise, mental well-being and burnout in Thai medical students in 2020–2021: an online cross-sectional survey

**DOI:** 10.1186/s12909-024-05843-y

**Published:** 2024-08-02

**Authors:** Dhachdanai Dhachpramuk, Suprapath Sonjaipanich, Supparat Theppiban, Supinya In-iw

**Affiliations:** Department of Pediatrics, Faculty of Medicine Siriraj Hospital, Mahidol University, Bangkok, Thailand

**Keywords:** Burnout, Depression, Exercise, Medical students, Mental well-being

## Abstract

**Introduction:**

Within the Thai medical curriculum, its rigorous education framework, demanding schedules and high academic standards can contribute to psychological distress. Regular physical activity has consistently shown positive effects on mental health. The aim of the study was to investigate the association between exercise and psychological well-being, including depression, anxiety, and burnout, in Thai medical students, and factors related to insufficient exercise and depression.

**Methods:**

The cross-sectional study was conducted among medical students in the university hospital during 2020–2021. Participants completed self-administered questionnaires consisting of demographic data, Godin-Shephard Leisure-Time Physical Activity Questionnaire, depression screening (9Q), Thai General Health Question-28 (4 domains: somatic symptoms, anxiety and insomnia, social dysfunction, severe depression), and Maslach burnout inventory (Emotional exhaustion (EE), Depersonalization (DP), Reduced Personal Achievement (rPA).

**Results:**

Of the 404 participants, 50.5% were women, the mean age (SD) was 21.06 (1.8) years, and 52% were in clinical years. The prevalence of insufficient exercise was 59.6%, depression (30.2%), somatic symptoms (27.7%), anxiety (30.7%), insomnia (89.4%), social dysfunction (89.4%), high level of EE (32.4%), DP (21%), and rPA (56.7%). Insufficient exercise was associated with moderate to severe depression (OR 2.89, 95% CI 1.16–7.25), anxiety and insomnia (OR 1.56, 95% CI 1.01–2.43), social dysfunction (OR 2.51, 95% CI 1.31–4.78), burnout in part due to high rPA (OR 2.4, 95% CI 1.4–4.13), and study in clinical years (OR 1.91, 95% CI 1.28–2.87). After adjusted significant factors, only studying in the clinical year, social dysfunction, and burnout in part of rPA were related to insufficient exercise.

**Conclusions:**

High rates of insufficient exercise, psychological challenges, and burnout were prevalent among medical students. To effectively address these issues, medical school should advise students to participate in regular exercise, promoting mental well-being and healthier lifestyles.

**Supplementary Information:**

The online version contains supplementary material available at 10.1186/s12909-024-05843-y.

## Introduction

Mental health challenges, including depression, anxiety, and burnout syndrome, have seen an increase in prevalence on a global scale, particularly among individuals within the medical field [1]. A study highlighted that medical trainees, which encompass medical students, residents, and fellows, exhibited significantly elevated rates of mental health disorder, with occurrences being eight times for generalized anxiety disorder and five times for major depressive disorder which were higher than national averages [2]. Furthermore, the systematic review and meta-analysis focused on undergraduate medical trainees, revealing mental health problems and seeking psychiatric intervention [3]. Similarly, the previous studies among medical students demonstrated high prevalence rates of depression, mental disorders, burnout, sleep problems [4]. Among Thai medical students at a northern medical school, 11.1% had depression. Similarly, in a southern medical school, a 21.1% depression diagnosis rate was reported, with 12.5% reporting suicidality [5, 6]. Moreover, one-third of fourth-to sixth-year medical students had poor mental health, while over half of them reported experiencing significant emotional exhaustion, despite the majority perceiving themselves as possessing a high degree of personal accomplishment [7]. These findings were consistent among six-year medical students across three medical schools [8]. It is noteworthy that both depression, mental health disorder, and burnout in medical students exceeded those within the general population [9]. The impact of these conditions on medical performance and attitude was also noted [10, 11].

Exercise emerged as a potential avenue for enhancing mental well-being and performance [12]. The effects of exercise on mental health are attributed to its influence on neurotransmitters like noradrenaline, serotonin, and insulin-like growth factor-1 [13]. A comprehensive meta-analysis affirmed the efficacy of both aerobic and strength training exercises in alleviating depressive symptoms [14]. Medical students engaging in 30-minute fitness classes experienced diminished perceived stress and notable improvements in physical, mental, and emotional well-being compared to those who exercised individually or irregularly [14]. A study involving American obstetric residents revealed that regular exercise contributed to a reduction in health problem reporting. Of these residents, more than half had experienced mental health issues, and exercise emerged as an intervention for symptom relief [15]. Correlations were also established between self-reported mental health status among medical students and engaging in vigorous and regular physical exercise [16]. Consistent with these outcomes, two additional studies underscored the positive impact of regular exercise on medical students’ physical, mental, emotional well-being, and overall quality of life [16, 17].

Explicitly investigating the relationship between exercise and mental well-being in Thai students is crucial due to the absence of previous research on the topic, particularly within the context of Thai medical students. Despite conducting a thorough literature review, no studies have been found that specifically examine the link between exercise, psychological well-being, and burnout among Thai medical students. Therefore, the primary objective of this study was to investigate the association between exercise and psychological well-being, including depression, anxiety, and burnout, in a Thai medical student population. The secondary objective was to study factors related to insufficient exercise and depression. The authors hypothesized that medical students who participated in regular exercise would have a lower rate of depression, anxiety, and burnout.

## Methods

The institutional review board committee in Faculty of medicine Sirriaj hospital, Mahidol University, approved the study. All experiments were performed in accordance with relevant guidelines and regulations. Medical students, in the 2020–2021 academic years were invited to participate in the study. Faculty of Medicine Sirriaj hospital is one of Thailand’s oldest and largest hospitals. It has the highest number of patients and medical trainees among all medical schools in the country. Exclusion criteria were medical students with diagnoses of mental illnesses as major depressive disorder, anxiety disorder, and other psychiatric conditions. The sample size for this cross-sectional analysis was determined using a 52.8% proportion of insufficient exercise from the previous study [18], with a 95% confidence interval and a 5% margin of error. It was determined that a minimum of 383 participants was required for this study.

The electronic informed consent was obtained after explaining the study process to the participants following the hybrid lecture in the classrooms. Participants are recruited by scanning QR codes on posters or images. Prior to accessing the online questionnaires and informed consent forms within the QR codes using Google Forms, researchers provide information about the research objectives to medical students during various classroom sessions, without any academic impact. Confidentiality is maintained to ensure anonymity, and participation is voluntary. The participants were not obligated to provide their signatures, and they maintained the freedom to withdraw from the research at any point. Approximately 30% of respondents participated in the study, a proportion closely aligning with the calculated sample size.

Participants completed self-administered online questionnaires providing demographic data, Thai version of Godin-Shephard Leisure-Time Physical Activity Questionnaire (GSLTPAQ), 9 questions (9Q), Thai version of the Maslach Burnout Inventory-general survey (MBI), and Thai general health question 28 (Thai GHQ-28).

-GSLTPAQ was used to assess the level of exercise that was calculated with the intensity and frequency scores. The scores were categorized into in three levels; less than 14 units indicated insufficient exercise, 14–23 units as moderately active exercise, and more than 23 units signified active exercise [19]. Sensitivity and specificity values of Godin-Shephard Leisure-Time Physical Activity Questionnaire were 75.3 and 58.5%, respectively. Cronbach’s alpha coefficients the scale in adolescents was 0.84 [20]. Spearman correlation coefficient (r_s_) between the Thai version and the English version of the GSLTPAQ was 0.95 [21].

The 9Q Thai version questionnaire consists of nine questions, each with rating scales ranging from 0 to 3 to indicate the increasing frequency of depressive symptoms. The optimal cutoff for 9Q scores is 7 or higher, with Cronbach’s alpha coefficients ranging from 0.78 to 0.82, a sensitivity of 86.15%, and a specificity of 83.12%. Scores ranging from 7 to 12 are defined as mild, 13 to 18 are classified as moderate, and scores of 19 or higher indicate severe [22].

The Thai GHQ-28 questionnaire assessed psychological well-being. This instrument displayed strong reliability and validity, with Cronbach’s alpha coefficients ranging from 0.86 to 0.95, and sensitivity and specificity ranging from 78.1 to 85.3% and 84.4–89.7%, respectively. It comprised 28 questions, categorized into four sections: somatic symptoms, anxiety and insomnia, social dysfunction, and severe depression. Each question had rating scales from 0 to 3 for the intensity of symptoms. Scores of more than 5 points for each part were interpreted as poor quality of life [23].

The Thai version of the Maslach Burnout Inventory (MBI), as translated by Sammawart S., underwent validation by experts and assessment of its reliability through the application of Cronbach’s alpha coefficient. The results of this analysis were segmented into three sections based on Cronbach’s alpha coefficients: Emotional Exhaustion (EE) 0.92, Depersonalization (DP) 0.66, and Personal Accomplishment (PA) 0.65. The questionnaire consisted of 22 questions in which the interpretation of each section employed distinct rating scales, categorizing participants into mild, moderate, and severe degrees. EE measured feelings of being emotionally overextended and exhausted by their study (Scores 0–16 are defined as mild, 17–26 as moderate, ≥ 27 as severe). DP an unfeeling and impersonal response toward patients (Scores 0–6 are defined as low, 7–12 as moderate, > 13 as high). PA measures feelings of competence and successful achievement in their work (Scores > 39 defined as low, 32–38 as moderate, and 0–31 as high) [24].

### Statistical analysis

Demographic data were presented using numbers and percentage. The analytical focus included a range of variables, comprising demographic data, exercise categories, anxiety levels, depression, general health assessment, and burnout scores. The association between categorical variables was evaluated with Chi-square tests and Fisher’s exact test, where appropriate. A multivariable binary logistic regression model was used to examine factors related to insufficient exercise and to assess factors associated with depression. Results were presented in the form of odds ratios (OR) with 95% confidence intervals (95% CIs). We entered all significant results into the final model, adjusting for age and gender. *P*-values less than 0.05 are considered statistically significant with 80% power. We used SPSS statistical software, version 18 (SPSS, Inc., Chicago, IL, USA) for all analyses.

## Results

There were 404 participants who voluntarily participated in the study, and all completed the questionnaires. Of these, 50.5% were women. The average age was 21.06 ± 1.8 (range 17–26). Of the 404 participants, 194 (48%) were in their preclinical years, while 210 (52%) were in their clinical years. Regarding exercise, running was first rank for 44.1% of participants, followed by gym workout at 28.5%. More than half of the medical students (59.6%) did not meet recommended exercise levels. On average, participants spent 1.29 h exercising and 3.49 h using social media. (Tables 1 and 2) The prevalence of depression among medical students in the study was 30.2%, while burnout rates were high, with 64.3% reporting moderate to severe emotional exhaustion, and 82.4% facing moderate to severe reduced personal achievement. Social dysfunction affected 89.4%, and one third experienced anxiety and insomnia. Additionally, 59.4% reported poor sleep. (Table 3)

**Table 1:** Demographic data of participants (*n* = 404)

Demographic		Mean (± SD)	
Age (years) (range)		21.06 (1.8) (17–26)	
Average grade points		3.59 (0.33)	
Social media use (hours)		3.49 (2.24)	
Total sleep duration (hours)		6.35 (1.1)	
Happy scores		7.29 (1.25)	
**Item**		**Numbers**	**Percentage (%)**
Gender	Female Male	204 200	50.5 49.5
Academic year of medical students	1st year 2nd year 3rd year 4th year 5th year 6th year	59 90 45 60 86 64	14.6 22.3 11.1 14.9 21.3 15.8
Body mass index	< 18 (kg/m^2^) 18–23 (kg/m^2^) > 23 (kg/m^2^)	35 277 92	8.6 68.6 22.8

**Table 2:** Characteristics of exercising among medical students (*n* = 404)

Characteristics		Mean (± SD)	
Time spending on exercise (hours/day)		1.29 (0.77)	
Number of days participated in exercising (day)		4.07(1.63)	
		**Numbers**	**Percentage (%)**
Types of exercise	Running Workout in gym Badminton Yoga Swimming Basketball Volleyball Tennis Football	178 115 65 62 41 23 16 12 11	44.1 28.5 16.1 15.3 10.1 5.7 4 3 2.7
Duration of exercise	< 30 min/day 31–60 min/day > 60 min/day	190 36 178	47.0 8.9 44.1
Godin-Shephard Leisure-Time Physical activity (GSLTPA)	Active exercise Moderate exercise Insufficiency exercise	99 64 241	24.5 15.8 59.6

**Table 3:** Characteristics of psychological well-being among medical students (*n* = 404)

Characteristics		Numbers	Percentage (%)
Self-reported psychological problems		306	75.7
Self-reported mental health issues		98	24.3
Self-reported depression		27	6.7
Self-reported suicidal ideation		25	6.19
Self-reported poor sleep		240	59.4
Self-reported good quality of life		161	39.9
Depression screening	No depression Mild depression Moderate to severe depression	282 92 30	69.8 22.8 7.4
General health question	Somatic symptom Anxiety and insomnia Social dysfunction Severe depression	112 124 361 54	27.7 30.7 89.4 13.4
Maslach burnout inventory: Emotional exhaustion	low level moderate level high level	144 129 131	35.6 31.9 32.4
Maslach burnout inventory: Depersonalization	low level moderate level high level	203 116 85	50.2 28.7 21.0
Maslach burnout inventory: Reduced personal accomplishment	low level moderate level high level	71 104 229	17.6 25.7 56.7

The mental health problems associated with insufficient exercise were moderate to severe depression (*p* = 0.018), anxiety and insomnia (*p* = 0.047), social dysfunction (*p* = 0.04), and burnout, in part due to reduced personal achievement (*p* = 0.006). Additionally, academic year of medical students (*p* = 0.001), self-reported poor quality of life (*p* = 0.001) and older than 20 years (*p* = 0.010) were more likely to be insufficient exercise significantly. According to mental well-being, medical students who feel depressed were more likely to burnout in all parts (*p* = 0.01, *p* < 0.001, *p* = 0.032), and reported poor quality of life (*p* = 0.006).

The multivariate analysis in Tables 4 and 5 shows that medical students who had insufficient exercise were more likely to have moderate to severe depression (OR = 2.89, 95%CI 1.16–7.25), anxiety and insomnia (OR = 1.56, 95%CI 1.01–2.43), social dysfunction (OR = 2.51, 95%CI 1.31–4.78), severe depression (OR = 1.91, 95%CI 1.01–3.58), high rPA (OR = 2.4, 95% CI 1.4–4.13), and study in clinical years (OR = 1.91,95%CI 1.28–2.87). After adjusting for all significant variables, social dysfunction, high rPA, and study in clinical years remained significant (OR 2.35,95% CI 1.21–4.57, OR 2.48, 95% CI 1.42–4.51,OR 1.94, 95% CI 1.28–2.94, respectively). Similar to those, depression was also related to studying in clinical years, having somatic symptoms, anxiety and insomnia, social dysfunction, and burnout except EE.

**Table 4:** Factors related to insufficient exercise in Thai medical students during 2020–2021 (*n* = 404)

Factors		OR	95% CI	aOR	95% CI
Age more than 20		1.69	1.13–2.53		
Gender		0.93	0.62–1.39		
Self-reported poor quality of life		2.59	1.48–4.55		
Moderate to severe depression		2.89	1.16–7.25		
Anxiety and insomnia		1.56	1.01–2.43		
Social dysfunction		2.51	1.31–4.78	2.35	1.21–4.57
Maslach Burnout Inventory: Reduced personal accomplishment	Low Moderate High	1 1.83 2.4	- 1.0-3.37 1.4–4.13	1 1.77 2.48	- 0.94–3.31 1.42–4.31
Clinical year of medical students (4th -6th )		1.91	1.28–2.87	1.94	1.28–2.94

95%CI = 95% confidential interval; aOR = Adjusted Odds Ratio; OR = Odds Ratio; 9Q = The 9Q Thai version questionnaire

**Table 5:** Factors associated with depression in Thai medical students during 2020–2021 (*n* = 404)

Factors		OR	95% CI	aOR	95% CI
Age more than 20		0.67	(0.43–1.02)		
Gender (Male)		0.81	(0.3–1.25)		
Self-reported poor quality of life		3.61	(2.19–5.95)		
Insufficient exercise		1.36	(0.88–2.11)		
Somatic symptoms		3.39	(2.56–4.56)	3.24	(1.73–6.06)
Anxiety and insomnia		4.00	(2.96–4.2)	4.06	(2.17–7.61)
Social dysfunction		2.79	(1.21–6.4)	3.23	(1.08–9.65)
Maslach burnout Inventory: Emotional exhaustion	Low Moderate High	1 4.29 6.97	- (2.28–8.07) (3.74–12.99)		
Maslach Burnout Inventory: Depersonalization	Low Moderate High	1 3.52 6.54	- (2.06-6) (3.69–11.61)	1 2.97 5.27	(1.56–45.65) (2.54–10.93)
Maslach burnout Inventory: Reduced personal accomplishment	Low Moderate High	1 1.73 3.28	(0.79–3.78) (1.64–6.58)	3.18 3.02	(1.18–8.57) (1.25–7.32)
Clinical year of medical students (4th -6th )		1.55	(1.02–2.39)	3.9	(2.1–7.14)

95%CI = 95% confidential interval; aOR = Adjusted Odds Ratio; OR = Odds Ratio

## Discussion

The study highlighted that medical students in the study who reported insufficient exercise demonstrated significant association with studying in clinical year, self-reported poor quality of life, moderate to severe depression, anxiety and insomnia, social dysfunction, and burnout in part of high reduced personal accomplishment. Our finding was similar to previous studies that physical exercise was inversely associated with mental health problems such as depression, anxiety, poor sleep quality, psychological distress and burnout [1, 25, 26]. Furthermore, a low level of exercise was associated with psychological distress such as burnout and depression among medical students [26].

In Thailand, medical schools provide a comprehensive six-year curriculum divided into two parts: the preclinical years (1st, 2nd, 3rd year) and the clinical years (4th, 5th, 6th year). In the 2nd year, the curriculum differs from the 1st and 3rd year of the preclinical years, emphasizing basic medical knowledge, laboratory classes, and frequent formative and summative assessments. Additionally, during the clinical years regarding to night shifts and the excessive workload, most medical students experience sleep deprivation, and a highly stressful environment. Consequently, they had less time available for regular exercise. More than half of the preclinical medical students managed their time on leisure activities, with the exception of 2nd -year medical students.

Insufficient exercise among medical students in the study was linked to increased levels of anxiety, insomnia, social dysfunction, and moderate to severe depression. This association can be attributed to the circumstances during the Covid-19 pandemic. Preclinical students studied from home, while clinical students faced social distancing measures and limited access to facilities, impacting their daily routines. Various studies have highlighted the pandemic’s adverse psychological effects on medical students [27–29]. They perceived themselves to be more stressed during clinical rotations and online education, which affected their learning and social life, ultimately leading to anxiety and depression [27–29]. Additionally, university students experienced reduced physical activity levels during the pandemic, coinciding with a higher prevalence of depression, anxiety, and stress [30, 31].

The prevalence of depression in the current study was 30.2%, which was higher than the previous study but similar to the results of the systematic review and meta-analysis in earlier studies [3, 4, 32]. Depressed medical students reported somatic symptoms, anxiety and insomnia, social dysfunction, poor quality of life, and burnout. Moderate and severe depression were associated with insufficient exercise, consistent with earlier researches indicating that reduced physical activity related to higher rates of negative emotional conditions such as depression, anxiety, and stress [31, 33]. Furthermore, medical students in the study experiencing depression was more likely to be studying in their clinical years and to report feelings of burnout. This result was similar to a previous study conducted in England, which stated that the majority of medical students who responded to the survey were exhausted [34]. In contrast to the previous study among medical students studying in Southern Thailand, it represented that pre-clinical year students experienced higher levels of depression and anxiety compared to clinical year students [32]. This trend may be attributed to various stressors, including the COVID-19 outbreak, online learning, virtual assessments, and reduced social interactions. During the COVID-19 pandemic, key stressors among Thai medical students included uncertainties regarding teaching modalities, concerns regarding potential system errors during exams, and the absence of clinical experience [35]. Practice during the clinical years was a significant factor related to psychological well-being, as well as over half of medical students reporting insufficient physical activity due to academic demands and shift work [36].

Burnout is a common issue among medical trainees, impacting their professional development, patient care, and personal well-being, including the occurrence of suicidal thoughts [37]. Burnout involved emotional exhaustion, depersonalization, and reduce personal accomplishment. Our study identified a significant correlation between reduced personal accomplishment and insufficient exercise, suggesting that students who felt less competent academically were less likely to engage in exercise. Furthermore, burnout in medical students were associated with unprofessional behavior, decreased patient care competence, and adverse effects on professional growth [37]. Strategies to improve burnout include increased social support, participating in recreational activities, hobbies, or exercise, to enhance coping skills. To reduce burnout and enhance mental health among medical students, it is imperative to promote healthy lifestyles that include regular exercise, adequate sleep, effective stress coping strategies, and fostering a positive learning environment.

The limitations of our study were considered. Firstly, it is important to note that our study design was cross-sectional, which means we could not establish causation or track longitudinal outcomes. Secondly, while our findings provide valuable insights into the specific group of the Thai medical students we studied, they may not represent all of the Thai medical students. Thirdly, the potential selection bias in our online survey suggests that participants who use social media might represent a specific subset of medical students, possibly seeking stress relief or having more available free time. This could impact the accuracy of mental health prevalence data in the study. As a result, we excluded individuals with mental disorders and other psychiatric conditions to reduce selection biases and provide more diverse demographic data.

Although the study possesses limitations, it contributes significantly to our understanding of the link between insufficient exercise and mental health among medical students. This underscores the importance of implementing targeted interventions. Key findings suggest the need for health guidance: monitoring quality of life, particularly during clinical years, and actively promoting healthy lifestyles characterized by regular exercise, adequate sleep, stress management skills, and a positive learning environment. Among medical students in Thailand, this study presented the association between insufficient exercise and mental well-being and burnout, enhancing our knowledge to prevent poor mental well-being or burnout with guidance not only on monitoring the quality of life, particularly among medical students in their clinical years, but also on emphasizing the fostering of healthy lifestyles. Such lifestyles are characterized by regular exercise, sufficient sleep, stress management skills, and a positive learning environment.

The recommendation for further study should include measuring the intensity of exercise, either by assessing cardiorespiratory fitness using a cardiopulmonary exercise test to measure percentages of maximal oxygen uptake (VO2max) or heart rate monitoring (HR), or by using metabolic equivalents to assess exercise intensity and estimate the energy expenditure of physical activities. Moreover, to gain a more comprehensive understanding, we advocate for the implementation of a multi-center cohort study that investigates the relationship between exercise and mental well-being, encompassing a more diverse sample. Such a study would produce more robust and widely applicable findings, ultimately contributing to the betterment of the broader medical field.

## Conclusions

High rates of insufficient exercise, psychological challenges, and burnout prevailed among medical students. To effectively address these issues, medical schools should advise students to participate in regular exercise, which promote mental well-being and healthier lifestyles. Medical institutions must establish affirmative policies recommending active exercise to prevent burnout and encourage positive psychological outcomes. In addition, interventions to prevent burnout, such as coping strategies and self-motivation, should be integrated into the program as mandatory extracurricular activities.

### Electronic supplementary material

Below is the link to the electronic supplementary material.

Supplementary Material 1

## Data Availability

The datasets used and analyzed during the current study are available from the corresponding author upon reasonable request.
