# Supplementary material for: Exercise, mental well-being and burnout in Thai medical students in 2020–2021: an online cross-sectional survey
Source: BMC Med Educ. 2024 Aug 2;24:837. doi: 10.1186/s12909-024-05843-y (PMC11297641; doi:10.1186/s12909-024-05843-y)
Supplement: Supplementary file 1 — Supplementary Material 1 [file 12909_2024_5843_MOESM1_ESM.pdf]

For use by Supinya Iniw only. Received from Mind Garden, Inc. on February 28, 2024

Permission for Supinya Iniw to administer 500 copies within three years of February 28, 2024

Maslach Burnout Inventory™

MBI Forms and Scoring Keys:

Human Services - MBI-HSS
Medical Personnel - MBI-HSS (MP)
Educators - MBI-ES
General - MBI-GS
Students - MBI-GS (S)

License to Administer

By Christina Maslach, Susan E. Jackson, Michael P. Leiter,
Wilmar B. Schaufeli & Richard L. Schwab

Published by Mind Garden, Inc.
www.mindgarden.com

Important Note to Licensee

It is your legal responsibility to compensate the copyright holder of this work — via payment to Mind Garden — for reproduction or administration in any physical or digital medium, including online survey, handheld survey devices, etc.

You agree to track the number of reproductions or administrations, and to compensate Mind Garden for any usage in excess of the quantity purchased.

This license is valid for three years from the date of purchase.

This instrument, and any use thereof, is covered by U.S. and international copyright laws. For any further use or reproduction of the instrument, in whole or in part, contact Mind Garden, Inc.

MBI-Human Services Survey: Copyright ©1981 Christina Maslach & Susan E. Jackson.
MBI-Human Services Survey for Medical Personnel: Copyright ©1981, 2016 Christina Maslach & Susan E. Jackson.
MBI-Educators Survey: Copyright ©1986 Christina Maslach, Susan E. Jackson & Richard L. Schwab.
MBI-General Survey: Copyright ©1996 Wilmar B. Schaufeli, Michael P. Leiter, Christina Maslach & Susan E. Jackson.
MBI-General Survey for Students: Copyright ©1996, 2016 Wilmar B. Schaufeli, Michael P. Leiter, Christina Maslach & Susan E. Jackson.
All rights reserved in all media. Published by Mind Garden, Inc., www.mindgarden.com

For use by Supinya Iniw only. Received from Mind Garden, Inc. on February 28, 2024

Permission Letter

www.mindgarden.com

To Whom It May Concern,

The above-named person has made a license purchase from Mind Garden, Inc. and has permission to administer the following copyrighted instrument up to that quantity purchased:

Maslach Burnout Inventory forms: Human Services Survey, Human Services Survey for Medical Personnel, Educators Survey, General Survey, or General Survey for Students.

The license holder has permission to administer the complete instrument in their research, however, only three sample items from this instrument as specified below may be included in the research write-up, thesis, or dissertation. Any other use must receive prior written permission from Mind Garden. The entire instrument form may not be included or reproduced at any time in any other published material. Please understand that disclosing more than we have authorized will compromise the integrity and value of the test.

Citation of the instrument must include the applicable copyright statement listed below.
Sample Items:

MBI - Human Services Survey - MBI-HSS:
I feel emotionally drained from my work.
I have accomplished many worthwhile things in this job.
I don't really care what happens to some recipients.

Copyright ©1981 Christina Maslach & Susan E. Jackson. All rights reserved in all media.
Published by Mind Garden, Inc., www.mindgarden.com

MBI - Human Services Survey for Medical Personnel - MBI-HSS (MP):
I feel emotionally drained from my work.
I have accomplished many worthwhile things in this job.
I don't really care what happens to some patients.

Copyright ©1981, 2016 by Christina Maslach & Susan E. Jackson. All rights reserved in all media.
Published by Mind Garden, Inc., www.mindgarden.com

MBI - Educators Survey - MBI-ES:
I feel emotionally drained from my work.
I have accomplished many worthwhile things in this job.
I don't really care what happens to some students.

Copyright ©1986 Christina Maslach, Susan E. Jackson & Richard L. Schwab. All rights reserved in all media. Published by Mind Garden, Inc., www.mindgarden.com

Cont'd on next page

For use by Supinya Iniw only. Received from Mind Garden, Inc. on February 28, 2024

MBI - General Survey - MBI-GS:

I feel emotionally drained from my work.
In my opinion, I am good at my job.
I doubt the significance of my work.

Copyright ©1996 Wilmar B. Schaufeli, Michael P. Leiter, Christina Maslach & Susan E. Jackson. All rights reserved in all media. Published by Mind Garden, Inc., www.mindgarden.com

MBI - General Survey for Students - MBI-GS (S):

I feel emotionally drained by my studies.
In my opinion, I am a good student.
I doubt the significance of my studies.

Copyright ©1996, 2016 Wilmar B. Schaufeli, Michael P. Leiter, Christina Maslach & Susan E. Jackson. All rights reserved in all media. Published by Mind Garden, Inc., www.mindgarden.com

Sincerely,

Robert Most
Mind Garden, Inc.
www.mindgarden.com
